# Cardiovascular disease and risk factors in adults with diabetes mellitus in Hungary: a population-based study

**DOI:** 10.3389/fendo.2023.1263365

**Published:** 2023-09-14

**Authors:** Battamir Ulambayar, Amr Sayed Ghanem, Nóra Kovács, László Trefán, Marianna Móré, Attila Csaba Nagy

**Affiliations:** ^1^ Department of Health Informatics, Institute of Health Informatics, Faculty of Health Sciences, University of Debrecen, Debrecen, Hungary; ^2^ Department of Public Health and Epidemiology, Faculty of Medicine, University of Debrecen, Debrecen, Hungary; ^3^ Institute of Social and Sociological Sciences, Faculty of Health Sciences, University of Debrecen, Nyíregyháza, Hungary; ^4^ Coordinating Centre for Epidemiology, University of Debrecen, Debrecen, Hungary

**Keywords:** diabetes mellitus, cardiovascular disease, diabetes complication, cardiovascular risk factors, European health interview survey

## Abstract

**Introduction:**

Diabetes mellitus (DM) and cardiovascular disease (CVD) such as acute myocardial infarction, stroke, and coronary artery disease are highly prevalent conditions that are responsible for significant morbidity and mortality, particularly in Hungary. The conditions are attributed to identical risk factors, and individuals with DM are primarily susceptible to cardiovascular complications, which are the leading causes of death and disability in patients with DM. The objective of this study was to estimate the prevalence of CVD in individuals with DM and to investigate the association between potential risk factors and the presence of CVD among individuals with DM in a population-based sample.

**Methods:**

The study was based on data from three waves of the European Health Interview Surveys (EHIS) conducted in Hungary in 2009, 2014, and 2019.

**Results:**

The prevalence of CVD among patients with DM decreased during the study period and that socioeconomic factors, cardiometabolic risk factors including high blood pressure and high cholesterol, and depression are major contributors to CVD burden in patients with DM in Hungary.

**Discussion:**

Our findings suggest the importance of regular check-up for hypertension and hypercholesterolemia, better focus on socioeconomic status, as well as ongoing monitoring of mental health among patients with diabetes. Further research is needed to understand the potential causes behind the observed decrease in CVD prevalence.

## 1 Introduction

According to the International Diabetes Federation’s (IDF) report, the worldwide number of people affected by diabetes mellitus (DM) in 2021 is estimated to be 537 million individuals (1). This statement suggests that approximately 10% of the adult population suffers from DM. It is anticipated that the previously mentioned number will increase to 643 million by the year 2030, and further rise to 783 million by 2045 (1). A substantial part of the disease burden can be attributed to diabetes-related complications such as cardiovascular disease (CVD) including acute myocardial infarction (AMI), coronary artery disease (CAD) and stroke, being the major causes of death and disability among people with DM (2). CVD affects approximately 32.2% of individuals with T2DM and accounts for 50.3% of all deaths in patients with T2DM, with CAD and stroke remaining the major contributors (3). Furthermore, cardiovascular complications have a detrimental impact on the quality of life of patients with DM (4) and considerably contribute to the burden of CVD (5). Therefore, understanding the major risk factors for CVD complications and mitigating the risk of non-fatal or fatal CVD events among people with DM has of great importance in reducing the disease burden.

The relationship between DM and CVD is complex and includes a wide range of variables (6). There exists a considerable overlap in the risk factors associated with CVD and DM, such as elevated body mass index (BMI), hypertension, and hypercholesterolemia, smoking, physical inactivity, dietary habits or alcohol consumption (7). Additionally, other factors that amplify the effects of the afore-mentioned risk factors, such as education, income level, and access to medical services (8, 9), can contribute to poor glycaemic control are likely to increase the risk of CVD in patients with DM. Moreover, other comorbidities such as depression may contribute to a higher risk CVD events among individuals with type 2 diabetes (10). CVD complications among DM patients may be prevented by lifestyle modification and proper medication (11, 12). Additionally, achieving health equity for people with diabetes requires an understanding of how social and economic issues affect their health (13).

The early detection and evaluation of an individual’s understanding pertaining to risk factors associated with CVD and the early detection of CVD risks are of great importance. The implementation of suitable screening measures for individuals at risk beside more effective educational interventions on cardiovascular disease can improve their overall quality of life (14).

The prevalence of various forms of CVD and associated mortality rates in Hungary have shown a declining trend, however, the figures remain approximately 1.5 times greater than those observed in other member states of the EU. Moreover, CVD is one of the major reasons why Hungary lags behind the majority of EU nations in terms of the potential years of life lost and the life expectancy at 65 (15).

Our study aimed to determine the prevalence of cardiovascular diseases and modifiable cardiovascular risk factors and to explore the association between risk factors and CVD among adults with DM in Hungary using data from European Health Interview Survey (EHIS) 2009, 2014 and 2019.

## 2 Methods

### 2.1 Study design

In this study, we utilized the European Health Interview Survey (EHIS) data, a series of cross-sectional studies conducted in Hungary in 2009, 2014, and 2019 to develop accurate health indicators for EU member states. These studies were conducted to evaluate the general health status, risk factors affecting health, and patient satisfaction with healthcare services in the population. Under the supervision of Eurostat, data were collected using a standardized questionnaire and method, as well as an adequate sample that was representative of the population (16) for each year.

**Figure 1:**
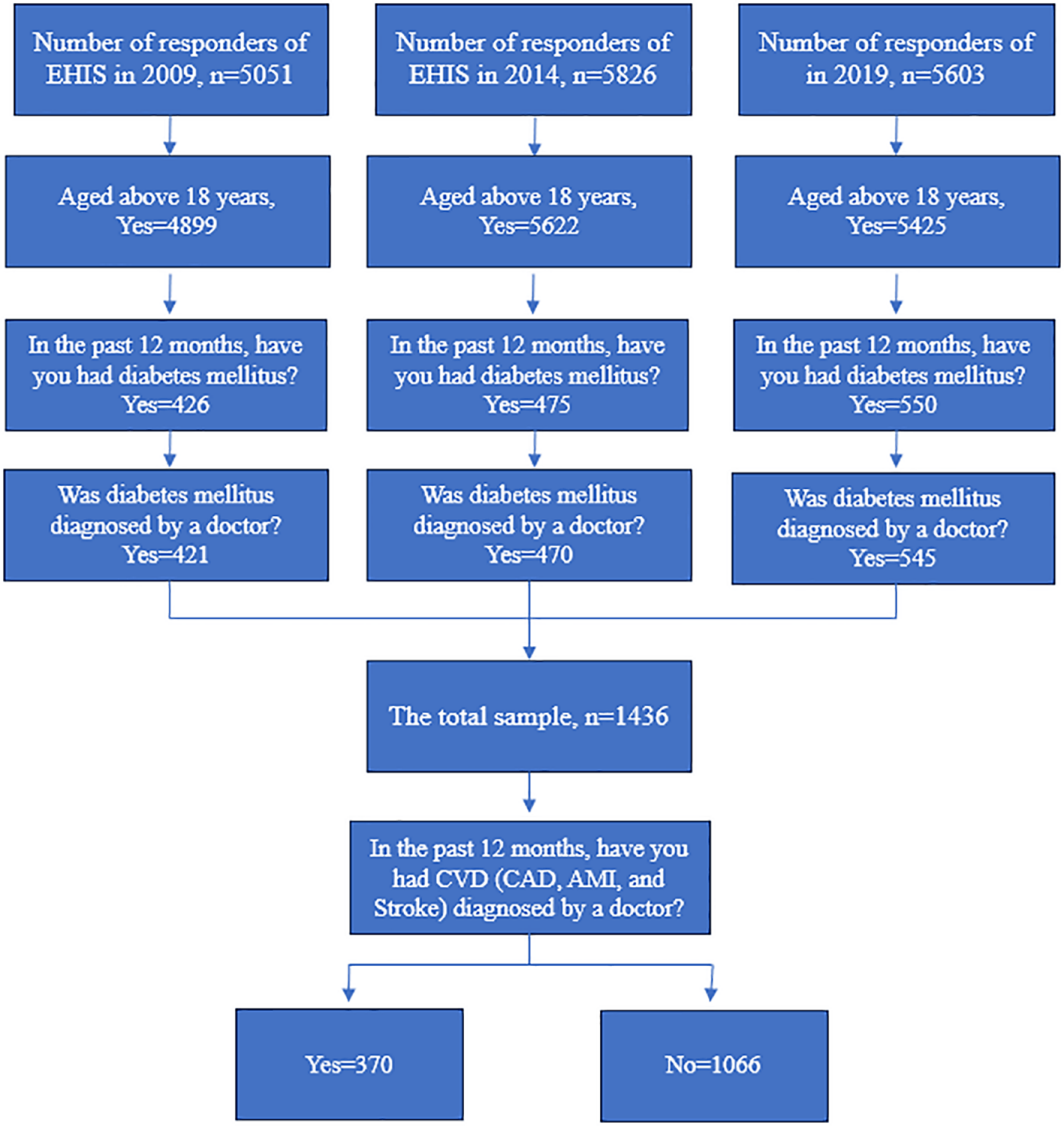
Flowchart of EHIS Initial and Final Samples from 2009, 2014, and 2019.

The EHIS data involved a sample size comprised of 5051 participants in 2009, 5826 in 2014, and 5603 in 2019 in Hungary, respectively. The final study sample consisted of N=421 (2009), N= 470 (2014) and N= 545 (2019) participants with DM. The study population included adults aged 18 years or older from Hungary. (**Figure 1**)

The research was carried out in adherence to the principles outlined in the Declaration of Helsinki and received approval from the Ethics Committee of the University of Debrecen (5609-2020) in compliance with Regulation 2016/679.

### 2.2 Variables

To establish a research sample, we implemented a two-step process to exclude data from patients who were not diagnosed with DM based on their self-reported history. Initially, the respondents who younger than 18 and answered negatively to the question regarding their status of DM within the past year were eliminated from the study. Subsequently, respondents who answered negatively to the question related to the physician’s diagnosis were excluded to ensure the validation of the prevalence. The type of DM is not indicated i’ the survey; hence all types of DM are included in the study. The presence of cardiovascular disease was identified based on two criteria: the respondent had any of the following conditions in the preceding one year: AMI, stroke, and CAD; and it was diagnosed by a doctor.

The analysis incorporated demographic and lifestyle factors such as gender, age (categorized as <65 or ≥65), the highest level of education (primary, secondary, tertiary), income level (low income, lower middle income, middle income, upper middle income, and high income), body mass index (categorized as normal (BMI<25), overweight (25≤BMI<30), and obese (BMI≥30)) (17), smoking status (classified as a smoker and non-smoker (including former smokers) and alcohol consumption (categorized as a heavy drinker, moderate, low, non-drinker) (18). Furthermore, the analysis included depression as comorbidity and variables regarding cardiometabolic risk factors as self-reported hypertension, and elevated blood cholesterol levels.

### 2.3 Statistical analysis

Pearson’s Chi-square test was applied to investigate the association between study variables. A multiple logistic regression analysis was performed in the pooled sample to investigate the association between potential risk factors and CVD in individuals with DM. Sampling weights were applied using the “svy” command in Stata to reflect population characteristics more accurately. The findings of the logistic regression were provided as odds ratios (ORs) and 95% confidence intervals (CI). STATA IC version 17.0 software package was used to perform all the statistical analyses (19).

## 3 Results

The prevalence of DM and CVD in patients with DM in Hungary over the three surveys is presented in **Table 1**. The data indicated a significant increasing trend in the prevalence of DM, from 8.3% (in 2009) to 9.7% (in 2019) (p=0.004), in contrast the prevalence of CVD among patients with DM showed a decreasing trend (from 33.9% to 20.4%; p<0.001). Among CVDs, AMI (p=0.003) and CAD (p<0.001) showed significant decreasing trend, and a non-significant slight decrease was observed in terms of stroke prevalence. (**Table 1**)

**Table 1:** Prevalence of DM and prevalence of CVD, AMI, stroke and CAD in patients with DM in EHIS 2009, 2014 and 2019.

Prevalence of conditions	Year of the survey						
	2009		2014		2019		p-value*
	n	%	n	%	n	%	
**DM in all participants**	421	8.3	470	8.1	545	9.7	**0.004**
**All CVD (including AMI, stroke, CAD) in patients with DM**	143	33.8	116	23.8	111	29.4	**<0.001**
** AMI in patients with DM**	55	13.1	38	7.9	40	6.9	**0.003**
** Stroke in patients with DM**	34	8.7	29	5.9	33	5.9	0.177
** CAD in patients with DM**	97	21.9	86	17.5	65	11.3	**<0.001**

*Bold values indicate statistical significance (p < 0.05) based on weighted Pearson’s chi-squared tests.

DM, diabetes mellitus; CVD, cardiovascular disease; AMI, acute myocardial infarction; CAD, coronary artery disease.

**Table 2** shows the characteristics of study participants with and without CVD. The results showed that 52.7% of patients with DM were female, 49.2% were aged over 65 years, and 52.2% have attained primary education. Additionally, 45.5% and 36.5% of DM participants were obese and overweight, 75% of them has high blood pressure, 37.8% had elevated blood cholesterol level, and 8.2% of them had depression in total pooled sample consisted of three waves of EHIS in Hungary. Individuals with CVD were significantly older compared with those without CVD (61.6% *vs*. 45.0%; p<0.001) and were more likely to have hypertension (84.9% *vs*. 71.5%; p<0.001), hypercholesterolemia (50.9% *vs*. 33.3%; p<0.001) and depression (14.2% *vs*. 6.1%; p<0.001). There was a statistically significant difference in the level of alcohol consumption (p=0.009) between diabetic participants with and without CVD. Furthermore, those with CVD was more likely to attain primary education (65.2% *vs*. 47.7%; p<0.001) and have lower income level (p=0.019). The BMI and smoking status were not differed significantly between study population according to CVD status. (**Table 2**)

**Table 2:** Characteristics of participants with a history of CVD among patients with DM.

Variable	Category	Participants with DM			
		total	with CVD	without CVD	p-value
			n (%)*	n (%)*	
Gender	Male	667 (47.3)	178 (47.9)	489 (47.1)	0.802
	Female	769 (52.7)	192 (52.1)	577 (52.9)	
Age group	<65	696 (50.8)	138 (38.4)	558 (55.0)	**<0.001**
	≥65	740 (49.2)	232 (61.6)	508 (45.0)	
BMI	Normal/underweight	242 (18)	58 (16.0)	184 (18.8)	0.339
	Overweight	522 (36.5)	143 (39.4)	379 (35.5)	
	Obese	658 (45.5)	168 (44.6)	490 (45.7)	
Smoking status	Non-smoker	1159 (81.2)	305 (82.9)	854 (80.6)	0.361
	Smoker	257 (18.8)	62 (17.1)	195 (19.4)	
Alcohol consumption	Never	613 (42.9)	187(50.6)	426 (40.2)	**0.009**
	Low	529 (37)	121(32.8)	408 (38.5)	
	Moderate	221 (15.7)	46 (12.9)	175 (16.7)	
	Heavy	60 (4.4)	15 (3.7)	45 (4.6)	
Hypercholesterolemia	No	888 (62.2)	178 (49.1)	710 (66.7)	**<0.001**
	Yes	548 (37.8)	192 (50.9)	356 (33.3)	
Hypertension	No	349 (25)	57 (15.1)	292 (28.5)	**<0.001**
	Yes	1087 (75)	313 (84.9)	774 (71.5)	
Depression	No	1317 (91.8)	316 (85.8)	1001 (93.9)	**<0.001**
	Yes	119 (8.2)	54 (14.2)	65 (6.1)	
Education level	Primary	740 (52.2)	238 (65.2)	502 (47.7)	**<0.001**
	Secondary	495 (33.7)	95 (25.2)	400 (36.7)	
	Higher	200 (14.1)	37 (9.6)	163 (15.6)	
Income level	Low	332 (23.8)	103 (28.8)	229 (22.0)	**0.019**
	Lower middle	322 (21.1)	86 (22.1)	236 (20.7)	
	Middle	293 (20.4)	74 (20.5)	219 (20.4)	
	Upper middle	293 (20.5)	73 (18.8)	220 (21.1)	
	High	196 (14.2)	34 (9.8)	162 (15.8)	

*unweighted numbers and weighted proportions. Bold values indicate statistical significance (p < 0.05) based on weighted Pearson’s chi-squared tests.

**Table 3** shows the results of the multiple logistic regression analysis. According to the multivariate model, females had a 40% lower likelihood of having CVD in comparison to males (OR 0.6; 95% CI 0.45 – 0.81). Those who were 65 and older were 2.13 times more likely to have CVD (OR 2.13; 95% CI 1.58 – 2.87). Hypertension (OR 1.53; 95% CI 1.05 – 2.22) and hypercholesteremia (OR 2.12; 95% CI 1.61 – 2.81) were significantly associated with CVD. The strongest association with CVD was observed in terms of depression (OR 2.62; 95% CI 1.69 – 4.04). Individuals with DM who have attained a secondary education level were 34% less likely to have CVD (OR 0.66; 95% CI 0.47 – 0.94), while those with higher education were 40% lower odds (OR 0.61; 95% CI 0.38 – 0.99) of having CVD compared to respondents with primary education. Regarding income level, low (OR 2.03; 95% CI 1.19 – 3.46) and lower-middle income (OR 1.79; 95% CI 1.05 – 3.06) showed significant positive association with CVD. The prevalence of CVD among patients with DM reduced in the years 2014 (OR 0.56; 95% CI 0.40 – 0.79) and 2019 (OR 0.53; 95% CI 0.36 – 0.77) compared to 2009. The model showed no significant association between CVD and lifestyle factors including BMI, smoking status, and alcohol consumption. (**Table 3**)

**Table 3:** Association between risk factors and the presence of CVD among individuals with DM by multivariate logistic regression analysis.

Characteristics		OR (95% CI)	p-value
Gender	Male (reference)		
	Female	**0.60 (0.45-0.81)**	**0.001**
Age group	<65		
	≥65	**2.13 (1.58-2.87)**	**<0.001**
BMI	Normal/underweight (reference)		
	Overweight	1.18 (0.78-1.79)	0.439
	Obese	0.94 (0.62-1.44)	0.79
Smoking status	Non-smoker (reference)		
	Smoker	1.03 (0.70-1.53)	0.871
Hypertension	No (reference)		
	Yes	**1.53 (1.05-2.22)**	**0.027**
Hypercholesterolemia	No (reference)		
	Yes	**2.12 (1.61-2.81)**	**<0.001**
Depression	No (reference)		
	Yes	**2.62 (1.69-4.04)**	**<0.001**
Alcohol consumption	Never (reference)		
	Low	0.78 (0.57-1.07)	0.118
	Moderate	0.66 (0.43-1.01)	0.058
	Heavy	0.59 (0.29-1.19)	0.141
Education level	Primary (reference)		
	Secondary	**0.66 (0.47-0.94)**	**0.021**
	Higher	**0.61 (0.38-0.99)**	**0.044**
Income level	High (reference)		
	Low	**2.03 (1.19-3.46)**	**0.010**
	Lower middle	**1.79 (1.05-3.06)**	**0.033**
	Middle	1.61 (0.94-2.74)	0.080
	Upper middle	1.59 (0.94-2.68)	0.085
Year	2009 (reference)		
	2014	**0.56 (0.40-0.79)**	**0.001**
	2019	**0.53 (0.36-0.77)**	**0.001**

Bold values represent the significant association (p<0.05). OR, Odds ratio; CI, Confidence interval. BMI, body mass index.

## 4 Discussion

We sought to identify potential factors that might be associated with CVD among people with DM and estimate the prevalence of CVD in the population with DM. Taken together, the results of our study suggest that variables such as older age, male gender, presence of cardiometabolic risk factors including hypertension and high cholesterol level, as well as depression have adverse impact on CVD complications among patients with DM.

The findings of our investigation demonstrated a decreasing trend in the prevalence of CVD in patients with DM in the Hungarian population. A study by Jung et al. revealed a noteworthy decrease in cardiovascular complications among individuals with diabetes in Korea between 2006 and 2013 (20). A comparable trend was noted in Australia between the years 2010 and 2019 (21). Harding et al. have determined that a declining worldwide tendency exists in terms of the prevalence of vascular complications of DM, such as AMI, stroke, and amputation (22). The collective findings of these studies indicate that although it appears to be a potential tendency towards a reduction in the frequency of CVD among individuals with diabetes, they continue to face significant burden due to cardiovascular complications on a global scale. Especially in developed countries, this pattern may be related to the quality and availability of healthcare services, the level of health education, and the new generations of drugs that improve glycaemic control, such as sodium glucose cotransporter 2 (SGLT2) inhibitors (23) glucagon-like peptide 1 (GLP-1) receptor agonist (24). The trend is also apparent in the findings of our research on the Hungarian population.

Prior research has indicated that women with DM exhibit increased susceptibility to CVD as a result of poor glycaemic management (25), in contrast to our findings. Conversely, certain studies have suggested that men with DM are at greater risk for CVD (26). In addition, a study conducted on patients with DM who were hospitalized due to heart failure concluded that gender does not significantly affect the occurrence of cardiovascular complications in patients with DM (27). The observed contradictory findings could potentially be attributed to the demographic profile of the participants involved in the studies. This variation could also demonstrate that the difference between genders depends on multiple other confounders such as genetics and body composition besides social and environmental factors in the form of occupational exposure, stress, and access to healthcare.

Numerous studies have demonstrated that the correlation between DM and CVD is primarily influenced by age, with an elevated likelihood of having cardiovascular complications in patients with DM as they age (28–30). Our results are consistent with this trend.

In addition, hypertension and dyslipidaemia, which were found to have a strong association with CVD in patients with DM, are not only the main metabolic symptoms (27, 31), but also the main indicators used to predict cardiovascular complications in patients with DM (32–34). This indicates that regular monitoring of blood pressure (BP) and blood lipid levels in diabetic patients and keeping them at appropriate levels is important to prevent cardiovascular complications.

One of the most prevalent co-morbidities in patients with diabetes is depression (35), which has been found to significantly increase mortality from all causes, including CVD (36). Our results showed a strong association between depression and CVD, which is in line with the pattern that depression is often co-morbid with DM and CVD.

The utilization of SGLT2 inhibitors has been proposed to lower cardiovascular risk in individuals diagnosed with T2DM by effectively decreasing hyperglycemia, BP, and body weight. SGLT2 inhibitors enhance cognitive processes as well by mitigating brain damage and cognitive deterioration (37, 38).

Regarding lifestyle factors, our study showed no significant relationship of CVD with BMI, smoking and alcohol consumption. The co-occurrence of obesity and CVD in patients with DM may be attributed to a complex mechanism of various factors, thereby presenting an opposing perspective to the conventional understanding of the relationship between obesity and CVD. A study of Chang’s et al. found a U-shaped relationship between the BMI and all-cause mortality among individuals with DM suggesting that those who were mild obese or overweight had a lower risk of mortality compared to individuals with normal BMI (39). Smoking is well-known risk factor for both DM and CVD, therefore patients with DM are recommended to quit smoking as a preventive measure prevent CVD (40). In contrast to prior research conducted in the same field (41), the findings of our study indicate that there is no statistically significant association within smoking status and CVD. Previous studies indicated that alcohol consumption may have varying impacts on cardiovascular risk among individuals with DM based on their level of consumption (42–45), however, our results indicate a lack of association between alcohol consumption and CVD in patients with DM. Our findings suggest that coexistence of CVD and DM makes people more likely to adhere to lifestyle-related recommendations.

Furthermore, results of this study indicate that lower socioeconomic status (lower level of education and lower income level) is associated with an increased likelihood of CVD, which aligns with the outcomes of recent studies among patients with DM (46–49). This can be explained by the fact that the cardiovascular complications of DM may be decreased by good glycaemic control (50), which can be achieved by effective health education (51).

Hyperglycemia significantly impacts cardiovascular outcomes, increasing rehospitalization risks in Ischemia and Nonobstructive Coronary Arteries (INOCA) patients and CVD risks in T2DM patients (52, 53). Hospitalized cardiovascular patients benefit from rigorous glycemic control (54, 55). Concurrent frailty, hypertension, and hyperglycemia in older adults has a significant impact on their physical well-being (56). Insulin resistance, measured by the Triglyceride and Glucose (TyG) index, has broad cardiovascular implications (57), and purinergic signaling offers potential therapeutic strategies for vascular complications (58).

### 4.1 Strengths and limitations

The study has several limitations. Data rely on self-reported responses from participants, which may be biased. Duration and severity of chronic diseases were not included in our analysis as they were not available in the surveys. Furthermore, the absence of available data related to specific biochemical parameters such as glycemia, HbA1c, creatinine, and albuminuria poses limitations for drawing deeper insights into the metabolic and renal status of the participants, which could potentially influence the outcomes related to diabetes and cardiovascular disease. However, the study also has several strengths including robust sampling methodology and a comprehensive data collection and processing protocol managed by Eurostat and the Hungarian Central Statistical Office. The results of the survey are representative to the Hungarian adult population. Additionally, the study employed a standardized questionnaire that is utilized across all European countries in three consecutive time periods.

## 5 Conclusion

This study provides insight into the prevalence of cardiovascular disease and associated risk factors among individuals with diabetes in the adult Hungarian population using three waves of EHIS. Our findings showed a declining trend in CVD prevalence among people with DM and suggested the importance of regular check-up for hypertension and hypercholesterolemia, as well as ongoing monitoring of mental health among patients with DM. The results also demonstrated the negative impact of socioeconomic status on DM complications, while lifestyle factors were not among the major contributors to the burden due to CVD among patients with DM. However, more research is needed to understand the potential causes behind the observed decrease in CVD prevalence.

## Data availability statement

The data analyzed in this study is subject to the following licenses/restrictions: The data presented in this study are available upon request from Hungarian Central Statistical Office who performed and supervised the data collection. Requests to access these datasets should be directed to Hungarian Central Statistical Office, www.ksh.hu/?lang=en.

## Ethics statement

The studies involving humans were approved by Ethics of Committee of the University of Debrecen (5609-2020). The studies were conducted in accordance with the local legislation and institutional requirements. Written informed consent for participation was not required from the participants or the participants’ legal guardians/next of kin in accordance with the national legislation and institutional requirements.

## Author contributions

BU: Conceptualization, Data curation, Formal Analysis, Writing – original draft. AG: Writing – original draft. NK: Conceptualization, Writing – review & editing. LT: Writing – review & editing. MM: Writing – review & editing. AN: Conceptualization, Formal Analysis, Supervision, Writing – review & editing.
